# CT-derived fractional flow reserve for prediction of major adverse cardiovascular events in diabetic patients

**DOI:** 10.1186/s12933-023-01801-y

**Published:** 2023-03-21

**Authors:** Ziting Lan, Xiaoying Ding, Yarong Yu, Lihua Yu, Wenli Yang, Xu Dai, Runjianya Ling, Yufan Wang, Wenyi Yang, Jiayin Zhang

**Affiliations:** 1 Department of Radiology, Shanghai General Hospital, Shanghai Jiao Tong University School of Medicine, #85 Wujin Rd, Shanghai, 200080 China; 2 Department of Endocrinology and Metabolism, Shanghai General Hospital, Shanghai Jiao Tong University School of Medicine, #85 Wujin Rd, Shanghai, China; 3 Institute of Diagnostic and Interventional Radiology, Shanghai Jiao Tong University Affiliated Sixth People’s Hospital, #600, Yishan Rd, Shanghai, China; 4 Department of Cardiology, Shanghai General Hospital, Shanghai Jiao Tong University School of Medicine, #85 Wujin Rd, Shanghai, China

**Keywords:** Diabetes, Coronary computed tomography angiography, Fractional flow reserve, High-risk plaque

## Abstract

**Objectives:**

To investigate the prognostic value of computed tomography fractional flow reserve (CT-FFR) in patients with diabetes and to establish a risk stratification model for major adverse cardiac event (MACE).

**Methods:**

Diabetic patients with intermediate pre-test probability of coronary artery disease were prospectively enrolled. All patients were referred for coronary computed tomography angiography and followed up for at least 2 years. In the training cohort comprising of 957 patients, two models were developed: model1 with the inclusion of clinical and conventional imaging parameters, model2 incorporating the above parameters + CT-FFR. An internal validation cohort comprising 411 patients and an independent external test cohort of 429 patients were used to validate the proposed models.

**Results:**

1797 patients (mean age: 61.0 ± 7.0 years, 1031 males) were finally included in the present study. MACE occurred in 7.18% (129/1797) of the current cohort during follow- up. Multivariate Cox regression analysis revealed that CT-FFR ≤ 0.80 (hazard ratio [HR] = 4.534, *p* < 0.001), HbA1c (HR = 1.142, *p* = 0.015) and low attenuation plaque (LAP) (HR = 3.973, *p* = 0.041) were the independent predictors for MACE. In the training cohort, the Log-likelihood test showed statistical significance between model1 and model2 (*p* < 0.001). The C-index of model2 was significantly larger than that of model1 (C-index = 0.82 [0.77–0.87] vs. 0.80 [0.75–0.85], *p* = 0.021). Similar findings were found in internal validation and external test cohorts.

**Conclusion:**

CT-FFR was a strong independent predictor for MACE in diabetic cohort. The model incorporating CT-FFR, LAP and HbA1c yielded excellent performance in predicting MACE.

**Supplementary Information:**

The online version contains supplementary material available at 10.1186/s12933-023-01801-y.

## Introduction

According to World Health Organization, the prevalence of diabetes have increased exponentially worldwide over the past few decades, from 108 million (4.7%) in 1980 to 425 million (8.5%) in 2017 [1]. Cardiovascular disease is one of the common complications of diabetes, including coronary artery disease (CAD), heart failure, arrhythmia and sudden cardiac death [1]. Compared with non-diabetic patients, diabetes shows a higher incidence of coronary atherosclerosis and greater probability of obstructive CAD [2, 3]. It is of clinical significance for precise risk stratification in diabetic patients to guide proper treatment strategy and therefore improve prognosis.

For invasive test, clinical evidence regarding the risk stratification in CAD patients with diabetes has been cumulated based on the plaque imaging by optical coherence tomography (OCT) or coronary microvascular function evaluated by pressure guidewire. In COMBINE OCT-FFR study, the thin-cap fibroatheroma detected by OCT was a strong predictor of major adverse clinical events (MACE) [4]. Meanwhile, microvascular dysfunction confirmed by index of microcirculatory resistance was also an independent predictor of MACE among diabetic patients with suspected CAD [5]. However, the invasiveness and high medical cost of the above tests significantly limit their clinical application in the general diabetic population.

For non-invasive imaging, coronary computed tomography angiography (CCTA) is recommended as the first-line test for CAD diagnosis [6, 7], which has high negative predictive value to safely rule out obstructive CAD. In addition to stenosis evaluation, CCTA is able to characterize high-risk plaque (HRP) features, such as low attenuation plaque (LAP), positive remodeling (PR), spotty calcification (SC) and napkin-ring sign (NRS) [8–10]. According to previous studies, higher stenotic grade as assessed by Coronary Artery Disease Reporting and Data System (CAD-RADS) [11] as well as the presence of HRP features are associated with poor prognosis [12, 13]. However, conventional CCTA data lacks functional evaluation regarding hemodynamic significance of coronary stenosis, which has become increasingly important in the diagnosis and treatment of CAD patients with diabetes over the years [14, 15].

Computed tomography angiography-derived fractional flow reserve (CT-FFR) is a non-invasive physiological test that enables functional assessment of flow-limiting stenosis based on CCTA data [16, 17]. This novel method is able to guide optimal treatment strategy with reduced unnecessary invasive procedures [18]. In multiple studies enrolling patients with suspected CAD, subjects with lesion-specific CT-FFR value > 0.8 have better prognosis than those with lesion-specific CT-FFR value ≦ 0.8 [19, 20]. However, there is a lack of evidence on the prognostic value of this promising approach in diabetic patients, which has high incidence of hemodynamic significant CAD as revealed by CT-FFR.

In light of the above findings, we hypothesized that CT-FFR might be a strong independent predictor for MACE in diabetic patients and has incremental value over other clinical and imaging parameters for risk stratification of diabetes. Thus, the aims of this study were to investigate the prognostic value of CT-FFR in patients with diabetes and to establish a risk stratification model for MACE by combining clinical risk factors, CT-FFR and HRP features.

## Materials and methods

### Study population

The hospital ethic committee approved this post-hoc analysis of a prospective cohort and all patients gave informed consents. We consecutively enrolled diabetic patients with intermediate pre-test probability of CAD (defined as pre-test probability between 15 and 85% according to updated Diamond–Forrester score [21]) from two hospitals from January, 2016 to December, 2019. All patients were referred for CCTA and followed up for at least 2 years. The exclusion criteria were: (1) severe renal dysfunction or allergic to CT contrast medium; (2) severe aortic stenosis or pulmonary hypertension; (3) any conditions that causing hemodynamic instability; (4) patients with history of coronary revascularization or myocardial infarction; (5) patients with non-ischemic cardiomyopathy disease or valvular disease; (6) impaired image quality of CCTA (insufficient to perform CT-FFR or plaque analysis); (7) patients underwent early revascularization (within 3 months after baseline CCTA) for lesions revealed by index CCTA; (8) lost clinical follow-up.

Participants in this study were divided into three separate cohorts: a training cohort, an internal validation cohort and an external test cohort. Specifically, patients from one hospital were randomly assigned to either the training cohort or the internal validation cohort at a 7:3 ratio. The external test cohort consisted of patients from another hospital.

### CCTA acquisition

A third-generation dual source CT (SOMATOM Force, Siemens Healthineers, Germany) or a 256-slice wide detector CT scanner (Revolution HD, GE Healthcare, USA) was used for scanning. Coronary Agatston calcium score (CACS) was firstly performed to assess the overall calcification burden of coronary vasculature. For CCTA acquisition, prospective ECG triggered technique was employed in all patients, covering 35%–75% of the R–R interval. Automated tube voltage and current modulation (CAREKv, CAREDose 4D, Siemens Healthineers, Germany; or KV Assist, Smart mA, GE Healthcare, USA) was applied to reduce radiation exposure. More details regarding acquisition parameters are given in the online appendix.

### CCTA-based plaque analysis

All CCTA data were transferred to an offline workstation (syngo.via, version VB20A, Siemens Healthineers, Germany) and the images with best quality were selected for manual diameter stenosis (DS) quantification. DS was defined as (reference diameter – minimal lumen diameter) / reference diameter. Patient-based stenosis severity was assessed according to the Coronary Artery Disease-Reporting and Data System (CAD-RADS) [11] while patients with CAD-RADS grade 3 or higher were considered having obstructive CAD.

Further plaque analysis was performed using a research software (Coronary Plaque Analysis, version 2.0, Siemens Healthineers, Germany), which allows semi-automatic plaque quantification [22, 23]. For all atherosclerotic lesions, four HRP features were characterized according to the following definitions: (1) PR, defined as any lesion with a remodeling index ≥ 1.1; (2) LAP, defined as any voxel < 30 HU within a coronary plaque; (3) SC, defined by an intra-plaque calcium < 3 mm in length that comprises < 90 degrees of the lesion circumference; (4) NRS, defined as a plaque core with low CT attenuation surrounded by a rim-like area of hyper-density [8–10, 24]. Any lesion with 2 or more HRP features were considered vulnerable plaque [25].

Two cardiovascular radiologists (with 3-years and 12-years experience of cardiac imaging), who were blinded to the clinical history and outcomes, independently analyzed the lesions. Any disagreement between two observers were resolved by consensus.

### CT-FFR simulation

CT-FFR simulation was performed on a research software package (Cta-Plus; version 2.0, Pulse Medical Imaging Technology, China) based on quantitative flow ratio (CT-QFR) technology. The diagnostic performance of this novel algorithm has been validated in previous studies using invasive FFR as the reference standard [26, 27]. The details regarding the computation and how onsite processing was performed are provided in the online appendix. Lesion-specific CT-FFR value was measured 1–2 cm distal to the lesion for all coronary stenosis on major epicardial vessels with diameter ≥ 2 mm [28]. Vessel-specific CT-FFR was defined as the CT-FFR value for the most distal lesion. For vessels without significant stenosis, the CT-FFR value was recorded at the most distal site where vessel diameter was ≥ 2 mm. The lowest vessel-specific CT-FFR value of major epicardial arteries was used for patient-based analysis and the presence of any vessel-specific CT-FFR ≤ 0.80 was considered hemodynamically significant.

Two cardiovascular radiologists (with 3-years and 12-years experience of cardiac imaging), who were blinded to the clinical history and outcomes, independently analyzed the lesions. The mean CT-FFR value of measurement by two observers was recorded for further analysis.

### Clinical follow-up and study endpoints

All enrolled patients were followed up for at least 2 years, or until the occurrence of MACE. Patients were followed-up every 6 months via outpatient visits. MACE was defined as all-cause mortality, cardiac death, non-fatal myocardial infarction, late revascularization (occurred three months after index CCTA), and rehospitalization due to heart failure or aggravated angina. The primary endpoint of this study was to determine the predictive value of CT-FFR for MACE in patients with diabetes. The secondary endpoint was to establish a risk stratification model for MACE by combining clinical risk factors, CT-FFR and HRP features.

### Statistical analysis

Continuous data were presented as mean ± standard deviation (SD) or median and interquartile range (IQR) depending on whether it conformed to a normal distribution, which was tested with Kolmogorov–Smirnov test. Categorical data were presented as absolute frequencies and proportions. Student's *t*-test or Mann–Whitney *U*-test (for two groups or ANOVA (for three groups) was used for continuous variables. Chi-square test or Fisher's exact test was used to compare the frequency distribution of categorical and binary data between subgroups, according to the size of data cells. Inter-observer and intra-observer agreement of CTA-derived parameters was assessed by intra-class correlation coefficient (ICC).

Cumulative incidence rates of MACE were estimated using Kaplan–Meier method and compared with the log-rank test, and using the following cut-off values: presence of HRP, CAD-RADS ≧ 3, and CT-FFR ≦ 0.8, whereas CACS groups were reclassified as CACS 0, CACS of 0 to less than 100, CACS of 100–400, and CACS greater than 400. In the training cohort, univariate and multivariate Cox proportional hazards regression models were used to analyze the prognostic value of various clinical and imaging parameters for MACE and to identify the independent predictors accordingly.

We built the base prediction model (model 1) with the inclusion of selected parameters (obstructive CAD, CACS, HRP, LAP, PR, SC and NRS, which were considered the known risk factors for MACE [8–11]. To demonstrate the incremental predictive value of CT-FFR, we used the nest model to build a new model (model 2) by overlaying CT-FFR on top of the base model. All models were adjusted for predefined sociodemo-graphic variables (age, sex, BMI) and cardiovascular risk factors (hypertension, diabetes, dyslipidemia, current-smoking, fast glucose, HbA1c). Incremental prognostic values of models were compared using Harrell’s C-statistics (C-index), log-likelihood test and time-dependent receiver operator characteristic (ROC) curve analysis. The goodness of fit of models was assessed by the calibration curve with Brier and score the Akaike information criterion (AIC). In addition, decision curve analysis was used to assess the clinical usefulness of the model by quantifying the net benefit at different threshold probabilities.

In the training set, a nomogram was developed based on model 2 (incorporating all significant independent predictors as revealed by multivariate Cox regression analysis) and obtained MACE probability estimates. This nomogram was further validated by C-index analysis in three cohorts. The score of each patient according to the nomogram was calculated and the median of the scores in the training cohort was selected as cutoff. It was further validated for the risk stratification performance in two validation cohorts. Kaplan–Meier curves with log-rank test were applied to compare patient survival between different groups. Two-sided *p* < 0.05 was considered statistically significant. Statistical analyses were performed using SPSS statistical package (version 26.0, IBM, Armonk, New York, USA) and the R statistical package (version 4.2.1).

## Results

### Patient characteristics

**Fig. 1:**
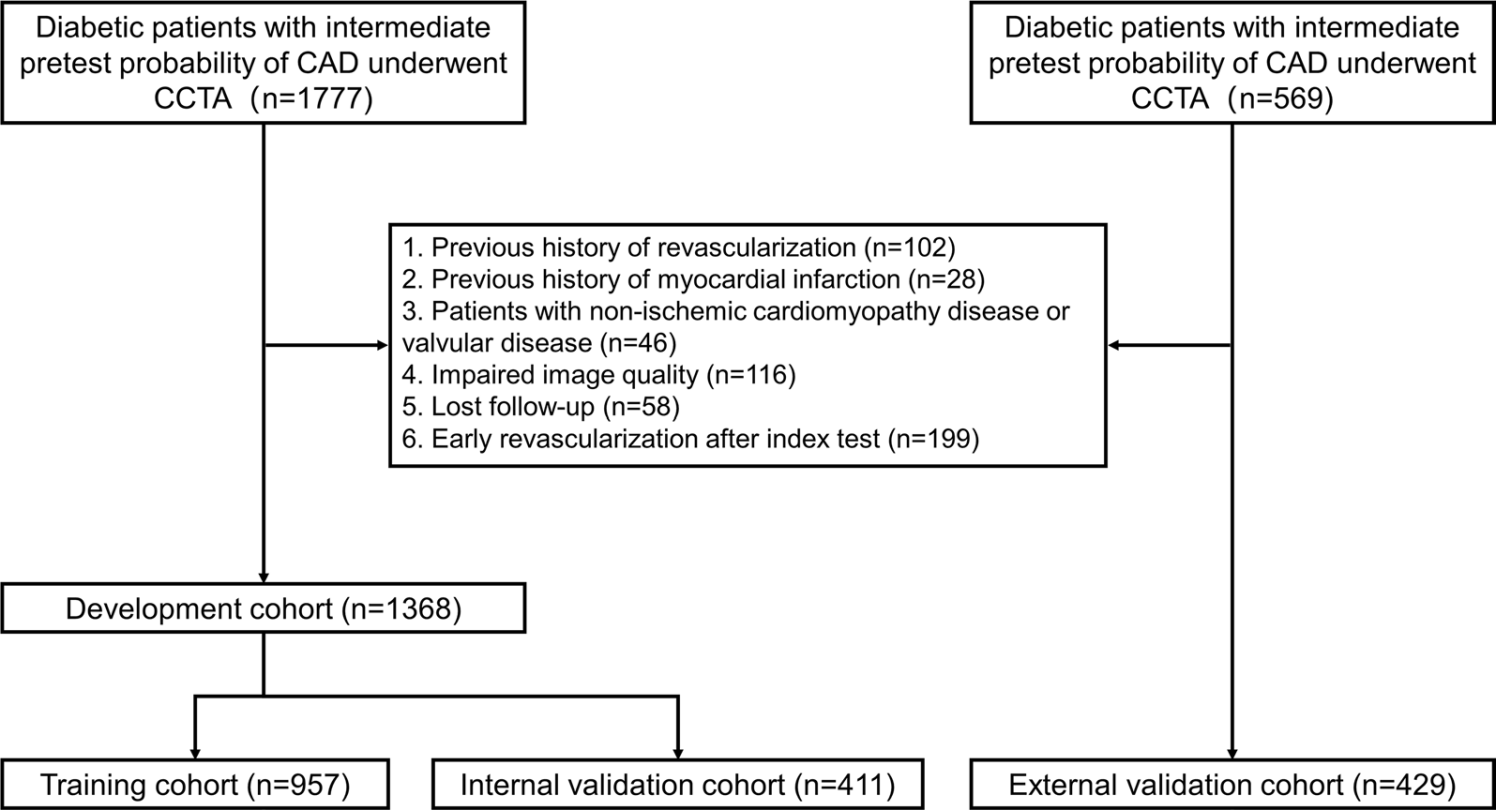
Flow chart of inclusion and exclusion criteria. CAD = coronary artery disease; CCTA = coronary computed tomography angiography;

A total of 2346 diabetic patients with intermediate pre-test probability of CAD were referred for CCTA from January, 2016 to December, 2019 and initially enrolled. Among them, 549 patients were excluded due to pre-specified criteria (Fig. 1). Eventually, there were 1797 subjects (mean age: 61.0 ± 7.0 years, 1031 males) included in the present study. As previously mentioned, patients were subsequently divided into training cohort (n = 957), internal validation cohort (n = 411), and external test cohort (n = 429). More details of demographic characteristics are given in Table 1 and Additional file 1: Table E1.

**Table 1:** Demographic data

	Training set	Internal validation set	*p*^#^	External test set	p*
	(n = 957)	(n = 411)		(n = 429)	
Age (years)	62.0 [55.0–69.0]	61.0 [54.0–69.0]	0.723	61.0 [54.0–68.0]	0.162
Males, n (%)	536 (56.0)	243 (59.1)	0.314	252 (58.7)	0.456
BMI, kg/m^2^	24.4 [22.5–26.8]	24.2 [22.3–26.4]	0.126	25.0 [22.6–27.3]	0.010
Course of diabetes (years)	10.0 [4.00–16.0]	10.0 [3.00–15.0]	0.147	10.0 [3.00–16.0]	0.310
Hypertension, n (%)	533 (55.7)	218 (53.0)	0.398	242 (56.4)	0.571
Dyslipidemia, n (%)	543 (56.7)	249 (60.6)	0.207	312 (72.7)	< 0.001
Current smoking, n (%)	300 (31.3)	122 (29.7)	0.584	128 (29.8)	0.766
Fast glucose (mmol/L)	7.00 [5.50–9.55]	6.80 [5.50–9.49]	0.484	7.67 [6.00–10.8]	< 0.001
HbA1c (%)	8.40 [7.20–10.0]	8.40 [7.10–10.1]	0.463	8.50 [7.20–10.3]	0.525
Radiation dose, mSv	1.92 [1.24–2.60]	1.86 [1.21–2.52]	0.375	2.49 [1.70–4.85]	< 0.001
CT-FFR ≤ 0.80, n (%)	73 (7.63)	41 (9.98)	0.182	26 (6.06)	0.103
Obstructive CAD, n (%)	286 (29.9)	127 (30.9)	0.756	90 (21.0)	0.001
CAD-RADS, n (%):			0.191		0.001
0	238 (24.9)	96 (23.4)		139 (32.4)	
1	219 (22.9)	75 (18.2)		101 (23.5)	
2	214 (22.4)	113 (27.5)		99 (23.1)	
3	172 (18.0)	75 (18.2)		59 (13.8)	
4A	103 (10.8)	43 (10.5)		27 (6.29)	
4B	9 (0.94)	8 (1.95)		2 (0.47)	
5	2 (0.21)	1 (0.24)		2 (0.47)	
CACS			0.415		0.800
0	427 (44.6)	167 (40.6)		191 (44.5)	
0–100	343 (35.8)	164 (39.9)		157 (36.6)	
100–400	120 (12.5)	55 (13.4)		54 (12.6)	
> 400	67 (7.00)	25 (6.08)		27 (6.29)	
HRP, n (%)	272 (28.4)	118 (28.7)	0.966	101 (23.5)	0.131
LAP, n (%)	277 (28.9)	120 (29.2)	0.977	97 (22.6)	0.034
PR, n (%)	505 (52.8)	226 (55.0)	0.487	225 (52.4)	0.706
SC, n (%)	58 (6.06)	27 (6.57)	0.814	23 (5.36)	0.759
NRS, n (%)	96 (10.0)	46 (11.2)	0.583	28 (6.53)	0.047
Microvascular complications, n (%)	694 (72.5)	288 (70.1)	0.392	330 (76.9)	0.072
MACE, n (%)	65 (6.79)	35 (8.52)	0.313	29 (6.76)	0.489

BMI, body mass index; CACS, Coronary Artery Calcium Scoring; CAD, Coronary artery disease; CAD-RADS, Coronary Artery Disease—Reporting and Data System; CT-FFR, computed tomography fractional flow reserve; HbA1c, hemoglobin A1c; HRP, high-risk plaque; LAP, low-attenuation plaque; MACE, major adverse cardiac events. NRS, napkin-ring sign; PR, positive remodeling; SC, spotty calcification

P^#^: the p between Training set and Internal validation set, P*: the p among the three cohorts

Values are mean ± SD, n (%), or median (IQR). SD, standard deviation

The dose-length products (DLP) for CCTA were 141.7 mGy × cm (93.6–215.4) mGy × cm and the mean effective dose of radiation for CCTA was 1.98 mSv (1.31–3.02) mSv when using 0.014 as the conversion coefficient. The median amount of contrast agent used for CCTA was 50 mL (45 mL, 55 mL). There were good Intra-observer and Inter-observer agreements in the measurement of all parameters (ICC > 0.75, *p* < 0.001 for all) (details shown in Additional file 1: Tables E3, E4).

### Clinical outcomes

All patients were followed-up for a median time of 3.14 years (2.58 years, 4.03 years). MACE occurred in 7.18% (129/1797) patients. Of these 129 patients who developed MACE, 97 patients were re-hospitalized due to heart failure (n = 3) or aggravated angina (n = 94), 18 patients experienced myocardial infarction, 11patients underwent late percutaneous coronary intervention, 3 patients died of cardiac death (n = 1) or noncardiac death (n = 2).

### Comparison of clinical and imaging parameters between patients with and without MACE

**Fig. 2:**
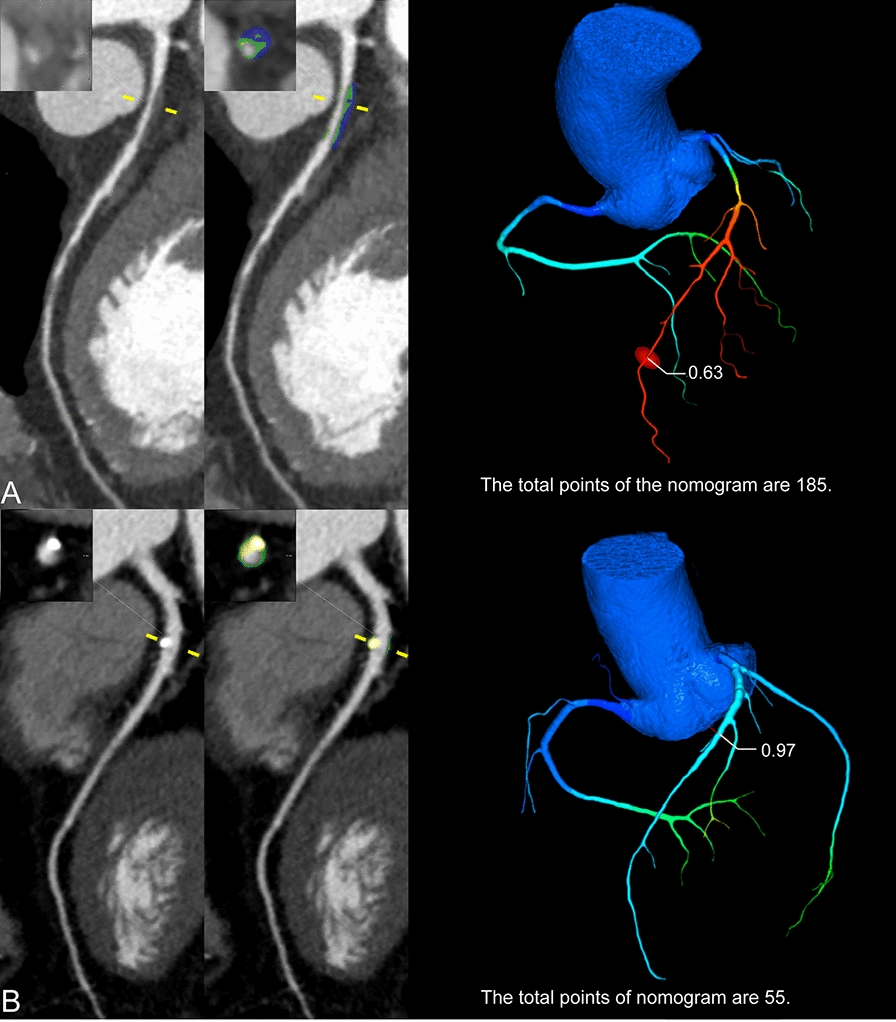
Representative cases of diabetic patients with and without MACE. (**A**) CCTA of a 63-year-old male with stable angina showed multiple obstructive stenosis at proximal and middle LAD. Plaque characterization revealed the presence of LAP for the proximal lesion (blue content) and the distal CT-FFR value was 0.63. The total points of the proposed nomogram were 185. This patient underwent late revascularization of proximal LAD lesion 2.29 years later due to aggravated angina symptom. (**B**) CCTA of a 79-year-old male with stable angina showed mild stenosis at proximal LAD. Plaque characterization revealed the absence of any HRP feature and the distal CT-FFR value was 0.97. The total points of the proposed nomogram were 55. The patient did not develop MACE at a follow-up of 2.72 years. CCTA = coronary computed tomography angiography; CT-FFR = computed tomography fractional flow reserve; HRP = high-risk plaque; LAP = low-attenuation plaque; LAD = left anterior descending; MACE = major adverse cardiac events

In both internal and external sets, patients with MACE had significantly higher incidence of having CT-FFR ≤ 0.8 or the presence of HRP compared to patients without MACE. Similar findings were also observed for obstructive CAD and higher CACS (Table 2; Fig. 2). Other clinical factors, such as age, gender, diabetes course, fast glucose level and HbA1c level, demonstrated discrepant results between different patient sets (Table 2). In addition, the medication did not show significant difference between patients with and without MACE in the training cohort, whereas more frequent use of some types of antihypertensive agents were noted in the internal and external validation cohorts (Additional file 1: Table E2).

**Table 2:** Clinical and imaging characteristics of patients with and without MACE

	Training set			Internal validation set			External test set		
	MACE (−)	MACE (+)	*p* value	MACE (−)	MACE (+)	*p* value	MACE (−)	MACE (+)	*p* value
	*N* = *892*	*N* = *65*		*N* = *376*	*N* = *35*		*N* = *400*	*N* = *29*	
Age (years)	62.0 [55.0–68.2]	65.0 [59.0–71.0]	0.012	61.0 [54.0–69.0]	67.0 [60.5–75.5]	0.002	61.0 [53.0–68.0]	64.0 [59.0–68.0]	0.103
Males, n (%)	493 (55.3)	43 (66.2)	0.115	224 (59.6)	19 (54.3)	0.668	235 (58.8)	17 (58.6)	1.000
BMI, kg/m^2^	24.4 [22.5–26.8]	24.9 [22.7–26.7]	0.764	24.2 [22.3–26.5]	24.5 [22.2–25.8]	0.833	25.0 [22.7–27.5]	24.0 [22.5–26.8]	0.521
Course of diabetes (years)	10.0 [4.00–16.0]	10.0 [6.00–17.0]	0.124	10.0 [3.00–15.0]	13.0 [5.00–17.5]	0.106	10.0 [3.00–16.0]	10.0 [5.00–15.0]	0.536
Hypertension, n (%)	489 (54.8)	44 (67.7)	0.059	193 (51.3)	25 (71.4)	0.036	220 (55.0)	22 (75.9)	0.046
Dyslipidemia, n (%)	508 (57.0)	35 (53.8)	0.720	229 (60.9)	20 (57.1)	0.799	291 (72.8)	21 (72.4)	1.000
Current smoking, n (%)	278 (31.2)	22 (33.8)	0.756	114 (30.3)	8 (22.9)	0.465	119 (29.8)	9 (31.0)	1.000
Fast glucose (mmol/L)	7.00 [5.46–9.50]	7.50 [5.96–9.86]	0.338	6.80 [5.52–9.50]	6.05 [5.29–9.34]	0.262	7.78 [6.02–10.9]	6.61 [5.40–9.02]	0.102
HbA1c (%)	8.30 [7.10–9.90]	9.40 [7.60–10.8]	0.029	8.40 [7.10–10.1]	8.50 [7.20–10.2]	0.737	8.50 [7.20–10.3]	8.90 [7.00–10.3]	0.940
CT-FFR ≤ 0.8, n (%)	46 (5.16)	27 (41.5)	< 0.001	27 (7.18)	14 (40.0)	< 0.001	17 (4.25)	9 (31.0)	< 0.001
Obstructive CAD, n (%)	240 (26.9)	46 (70.8)	< 0.001	102 (27.1)	25 (71.4)	< 0.001	72 (18.0)	18 (62.1)	< 0.001
CACS	2.56 [0.00–45.7]	87.5 [13.1–275]	< 0.001	5.69 [0.00–59.9]	47.9 [11.1–243]	< 0.001	2.25 [0.00–48.2]	59.9 [11.1–262]	< 0.001
HRP, n (%)	231 (25.9)	41 (63.1)	< 0.001	97 (25.8)	21 (60.0)	< 0.001	86 (21.5)	15 (51.7)	0.001
LAP, n (%)	234 (26.2)	43 (66.2)	< 0.001	98 (26.1)	22 (62.9)	< 0.001	84 (21.0)	13 (44.8)	0.006
PR, n (%)	450 (50.4)	55 (84.6)	< 0.001	195 (51.9)	31 (88.6)	< 0.001	200 (50.0)	25 (86.2)	< 0.001
SC, n (%)	49 (5.49)	9 (13.8)	0.013	22 (5.85)	5 (14.3)	0.068	20 (5.00)	3 (10.3)	0.197
NRS, n (%)	81 (9.08)	15 (23.1)	0.001	37 (9.84)	9 (25.7)	0.009	23 (5.75)	5 (17.2)	0.032
Microvascular complications, n (%)	645 (72.3)	49 (75.4)	0.695	261 (69.4)	27 (77.1)	0.446	305 (76.2)	25 (86.2)	0.317

BMI, body mass index; CACS, Coronary Artery Calcium Scoring; CAD, Coronary artery disease; CT-FFR, computed tomography fractional flow reserve; HbA1c, hemoglobin A1c; HRP, high-risk plaque; LAP, low-attenuation plaque; MACE, major adverse cardiovascular event; NRS, napkin-ring sign; PR, positive remodeling; SC, spotty calcification

Values are mean ± SD, n (%), or median (IQR). SD, standard deviation

**Fig. 3:**
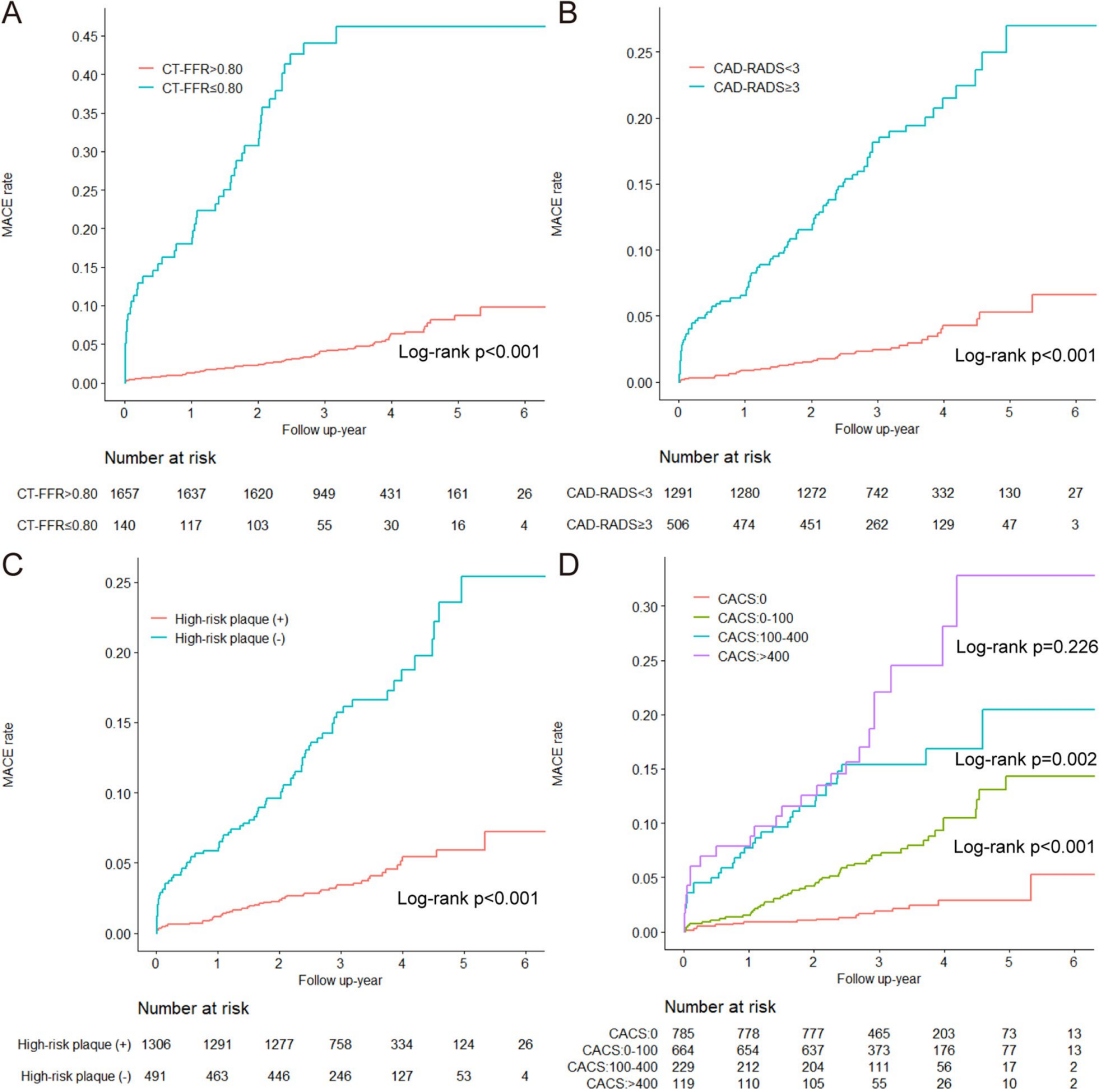
Kaplan–Meier curves for cumulative event rate of MACE according to (**A**) CT-FFR; (**B**) CAD-RADS classification; (**C**) HRP; (**D**) CACS. CACS = coronary artery calcium score; CAD-RADS = Coronary Artery Disease—Reporting and Data System; CT-FFR = computed tomography fractional flow reserve; HRP = high-risk plaque; MACE = major adverse cardiac event

According to Kaplan–Meier survival curves, patients with CT-FFR ≤ 0.8 or the presence of HRP had markedly higher MACE rate compared to patients without flow-limiting lesions or vulnerable plaques (Fig. 3). Similar findings were also noted for patients with obstructive CAD or CACS > 400.

### Development and validation of prediction models

**Fig. 4:**
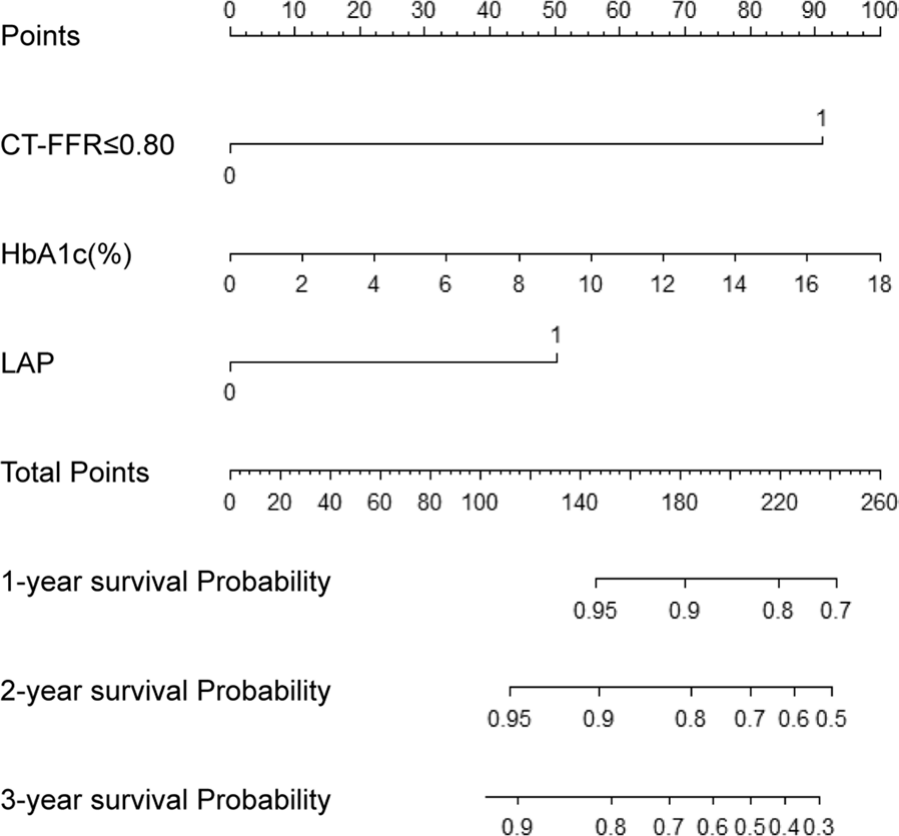
Nomogram for 1-, 2-, and 3-year probability of MACE. HbA1c = hemoglobin A1c; CT-FFR = computed tomography fractional flow reserve; LAP = low-attenuation plaque

In the training set, all clinical and imaging parameters were screened as potential predictors for MACE. According to univariate analysis, age, CACS, HbA1c, CT-FFR ≤ 0.80, CAD-RADS ≥ 3, HRP features and Alpha-glucosidase inhibitor were all predictors for MACE (*p* < 0.05), which were subsequently included into further multivariate Cox regression analysis. Multivariate Cox regression analysis found that CT-FFR ≤ 0.80 (HR = 4.534, *p* < 0.001), HbA1c (HR = 1.142, *p* = 0.015) and LAP (HR = 3.973, *p* = 0.041) remained the independent predictors for MACE. These three factors in model 2 were used to construct a nomogram at 1-, 2-, and 3-year to predict the probability of MACE (Fig. 4). The details of univariate and multivariable Cox regression analyses are presented in Table 3.

**Table 3:** Univariate and multivariable Cox regression analysis of clinical and imaging predictors for MACE in the training cohort

	Univariate analysis			Multivariate analysis 1*			Multivariate analysis 2*		
	HR	95%CI	p value	HR	95%CI	p value	HR	95%CI	p value
Age (per + 1 year)	1.034	1.010–1.059	0.006	1.016	0.991–1.042	0.214	1.020	0.994–1.048	0.131
Male	1.520	0.909–2.541	0.110						
BMI, (per + 1 kg/m^2^)	0.994	0.926–1.066	0.859						
Course of Diabetes (per + 1 year)	1.018	0.990–1.047	0.202						
Hypertension	1.682	1.000–2.829	0.050	1.202	0.700–2.063	0.504	1.078	0.621–1.869	0.790
Dyslipidemia	0.991	0.607–1.616	0.970						
Current smoking	1.042	0.623–1.744	0.875						
fast glucose (per + 1 mmol/L)	1.037	0.983–1.094	0.186						
HbA1c (per + 1%)	1.127	1.015–1.252	0.025	1.133	1.017–1.263	0.024	1.142	1.026–1.272	0.015
CACS (per + 1)	1.002	1.001–1.002	< 0.001	1.001	1.001–1.002	< 0.001	1.001	1.000–1.001	0.089
CT-FFR ≤ 0.8	10.865	6.627–17.814	< 0.001				4.534	2.468–8.330	< 0.001
CAD-RADS ≥ 3	6.109	3.579–10.429	< 0.001	2.192	1.117–4.299	0.022	1.856	0.932–3.697	0.078
Any HRP	4.530	2.737–7.499	< 0.001	0.567	0.146–2.206	0.413	0.375	0.090–1.564	0.178
Any LAP	5.075	3.035–8.485	< 0.001	3.667	1.031–13.048	0.045	3.973	1.060–14.893	0.041
Any PR	5.138	2.619–10.079	< 0.001	1.571	0.624–3.954	0.338	1.661	0.652–4.231	0.288
Any SC	2.667	1.319–5.395	0.006	1.046	0.492–2.225	0.908	0.907	0.419–1.961	0.804
Any NRS	3.104	1.739–5.540	< 0.001	1.311	0.681–2.524	0.418	1.209	0.624–2.344	0.573
Microvascular complications	1.250	0.710–2.200	0.439						
Insulin secretagogues	0.638	0.353–1.152	0.136						
TZDs	1.698	0.415–6.941	0.461						
Insulin	0.887	0.540–1.456	0.634						
Biguanides	0.925	0.568–1.507	0.755						
α-Glucosidase inhibitors	1.718	1.053–2.805	0.030	1.516	0.915–2.512	0.107	1.626	0.975–2.713	0.063
DPP-4 inhibitors	1.498	0.895–2.507	0.124						
SGLT-2 inhibitor	1.623	0.508–5.192	0.414						
GLP-1 RAs	1.402	0.439–4.474	0.568						
β-blockers	1.143	0.416–3.145	0.795						
ACEI	1.344	0.580–3.112	0.491						
ARB	1.106	0.648–1.888	0.712						
Ca2 + channel blockers	0.986	0.578–1.682	0.958						
Statins	1.678	0.896–3.141	0.106						

ACEI, angiotensin-converting enzyme inhibitors; ARB, angiotensin receptor antagonist; BMI, body mass index; CACS, Coronary Artery Calcium Scoring; CAD-RADS, Coronary Artery Disease—Reporting and Data System; CT-FFR, computed tomography fractional flow reserve; DPP-4, dipeptidyl peptidase 4; GLP-1 RA, glucagon-like peptide 1 receptor agonist; HbA1c, hemoglobin A1c; HRP, high-risk plaque; LAP, low-attenuation plaque; MACE, major adverse cardiovascular event; NRS, napkin-ring sign; PR, positive remodeling; SC, spotty calcification; SGLT-2, sodium-glucose cotransporter 2; TZDs, Thiazolidinedione drugs

^*^Multivariate analysis 1 was used for constructing model 1 and multivariate analysis 2 was used for constructing model 2

We further tested the predictive performance of established models for MACE in diabetes in internal validation and external test sets. The Log-likelihood test showed statistical significance between model 1 and model 2 in all the three cohorts (*p* < 0.001, respectively). In the training cohort, the C-index of model 2 was significantly larger than that of model 1 (C-index = 0.82 (95%CI = 0.77–0.87) vs. C-index = 0.80 (95%CI = 0.75–0.85), *p* = 0.021). Similar findings were revealed in internal validation and external test cohorts (Table 4). Moreover, the AUCs of model 2 for predicting 1- and 3-year MACE were significantly larger than those of model 1 in the training cohort. For the validation of the nomogram, the C-index was 0.80 (95% CI 0.75–0.86), 0.78 (95% CI 0.70–0.87) and 0.82 (95% CI 0.75–0.89) in the internal training, internal validation and external test cohorts respectively. The Kaplan–Meier curves in all three cohorts demonstrated that there were significant differences regarding the survival of patients in each risk group (Additional file 1: Fig. E1). The results were further validated in the internal validation and external test cohorts (Table 4). However, it is also notable that the C-index of the proposed model dropped to 0.621 (95% CI 0.45–0.80) in diabetic patients with CT-FFR value between 0.75 and 0.80.

**Table 4:** Prediction accuracy and risk reclassification of each model

Models	P Value by the Log-likelihood test	C-index (95%CI)	P Value	Time-dependent AUC		AIC	Brier score	
				1-year	3-year		1-year	3-year
Training set								
Model 1	< 0.001	0.80 (0.75–0.85)	0.021	0.82	0.82	801.08	0.011	0.026
Model 2		0.82 (0.77–0.87)		0.85	0.84	786.25	0.011	0.024
Internal validation set								
Model 1	< 0.001	0.80 (0.73–0.87)	0.022	0.80	0.84	384.64	0.025	0.039
Model 2		0.84 (0.77–0.90)		0.83	0.87	371.56	0.021	0.035
External test set								
Model 1	< 0.001	0.82(0.74–0.90)	0.027	0.89	0.82	328.27	0.017	0.031
Model 2		0.85(0.77–0.93)		0.92	0.87	313.06	0.015	0.025

AIC, Akaike information criterion; C-index, Harrell’s C-statistics

Model 1 included age, sex, BMI, hypertension, diabetes, dyslipidemia, current-smoking, fast glucose, HbA1c, Obstructive CAD, CACS, HRP, LAP, PR, SC and NRS

Model 2 = Model 1 + CT-FFR

**Fig. 5:**
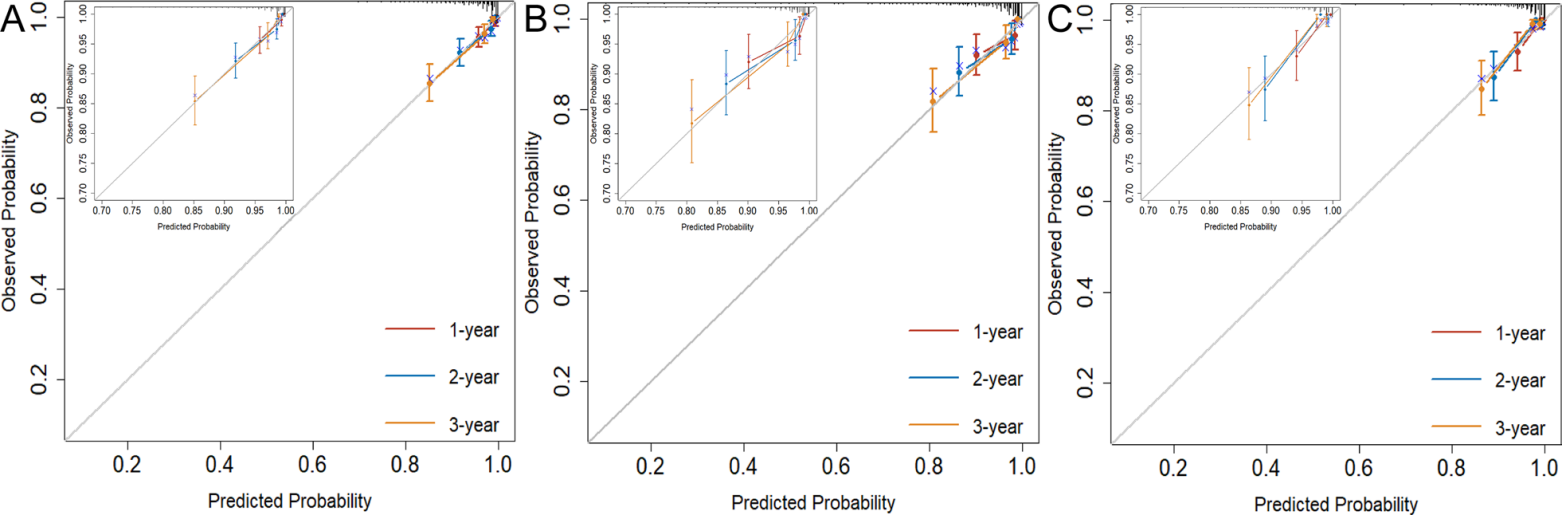
Calibration curves of the nomogram for 1-, 2-, and 3-year of MACE. Nomograms (**A**) for training cohort; (**B**) for internal validation cohort; and (**C**) for external validation cohort. MACE = major adverse cardiac event

Based on model 2, the calibration curves showed excellent consistency between prediction and observation in all three cohorts (Fig. 5). In addition, the model 2 outperformed model 1 with a lower Brier score for 1- and 3-year outcomes in the training, internal validation and external test cohorts (Table 4). The model 2 also had lower AIC results than did model 1 in all three cohorts (Table 4).

In the present study, the clinical benefits of model 2 were compared with model 1 in three cohorts by using the decision curve for 1- and 3-year events (Additional file 1: Fig. E2). Accordingly, model 2 provided incremental net benefits within a reasonable threshold probability compared to Model 1 in all three cohorts.

## Discussion

The major finding of the present study is to confirm CT-FFR as the strong independent predictor for MACE in patients with diabetes. Moreover, incorporating CT-FFR into risk stratification model provided incremental value over conventional clinical and anatomical parameters for prediction of MACE.

Patients with diabetes are two times more likely to develop MACE compared with those without diabetes [29]. Accurate risk stratification is a fundamental step to guide proper therapeutic decision [30]. Previous prediction models for MACE in diabetic patients are mainly based on clinical variables [31, 32], without taking the coronary imaging findings into consideration. However, previous studies have confirmed that diabetes is associated with higher incidence of vulnerable plaques, coronary calcification and higher atherosclerotic burden [33]. These pathological features are strongly related to unfavorable outcomes and can be non-invasively evaluated by CCTA. Therefore, it is of clinical importance to develop a prediction model incorporating clinical factors as well as coronary imaging features.

CCTA is a valuable non-invasive imaging modality to detect coronary atherosclerosis and follow-up plaque progression in patients with diabetes [34]. Previous studies using CCTA have demonstrated that obstructive stenosis and HRP features are two independent predictors for MACE in diabetic cohorts [35, 36] and help to enhance risk stratification. However, CT-FFR, as an advanced functional imaging approach, has not been investigated for its prognostic value in diabetes.

In the present study, we successfully developed a novel prediction model, consisting of both clinical variable and imaging parameters. According to multivariate Cox regression analysis, it was CT-FFR rather than obstructive CAD (CAD-RADS ≥ 3) that served as the independent predictor for MACE. This result could be ascribed to the advantages of CT-FFR over conventional stenotic extent to identify true flow-limiting lesions [37]. In this regard, the ischemic stenosis, which can be diagnosed by CT-FFR, tends to result in unrelieved symptom or late revascularization, despite of its stenotic severity. On the other hand, LAP was an independent imaging predictor for MACE in the present cohort. This HRP feature correlates to the large necrotic core in vulnerable plaque and is strongly associated with acute coronary syndrome [9]. It represents the high-risk lesion that is prone to rupture if not intensively treated. HbA1c was another independent predictor for MACE in the present cohort. Higher baseline HbA1c levels were found in this study. A previous study found that HbA1c levels at baseline were significantly associated with baseline plaque burden [38]. There is substantial evidence that increased chronic mean high blood glucose levels (usually HbA1c) are associated with a variety of diabetic complications including microvascular and macrovascular events [39, 40], particularly when levels are substantially elevated [41, 42]. Moreover, to test its generalizability, the current prediction model was further validated via an independent external dataset, which also exhibited excellent performance for predicting MACE. Therefore, the proposed model provided a comprehensive approach for cardiovascular risk stratification in diabetic patients with intermediate pre-test probability of CAD, who are the candidates for CCTA imaging as recommended by the present guidelines [6, 7].

In light of the above findings, the clinical implication of the present study lies in the following aspect. The application of CT-FFR is more favored than CT-based stenosis assessment for risk stratification. Compared to using CT-based anatomical evaluation, adding CT-FFR further improves the model performance for identifying diabetic patients at high risk of MACE. It consequently helps to guide appropriate therapeutic strategy and reduce unfavorable outcomes.

Despite the aforementioned promising results, the current study has several limitations. First, CT-FFR is a parameter that indicates the hemodynamic significance of epicardial coronary arteries. However, diabetes may also result in microvascular dysfunction (MVD), which can only be evaluated by other additional functional imaging modalities, such as cardiac magnetic resonance imaging [43]. Therefore, the risk of diabetic patients with MVD might be underestimated by the present model. In addition, the current cohort in majority consisted of diabetic patients with intermediate pre-test probability of CAD. Thus, the model cannot be directly applied to other diabetic cohorts, either with low or high pre-test probability. Finally, the nomogram score had significantly decreased predictive performance in diabetic patients with CT-FFR value between 0.75 and 0.80. It can be ascribed to the impaired diagnostic accuracy of CT-FFR for “grey-zone” lesions [44].

In conclusion, vessel-specific CT-FFR was a strong independent predictor for MACE in diabetic cohorts. The model incorporating CT-FFR, LAP and HbA1c yielded excellent performance in predicting MACE.

## Supplementary Information

**Additional file 1.** **Figure E1.** Kaplan–Meier curves for patients in the low- and high-risk groups in training cohort (A), internal validation cohort (B), and external validation cohort (C). **Figure E2.** Decision curves of prediction model for (A) 1-year MACE in the training cohort; (B) 3-year MACE in the training cohort internal; (C) 1-year MACE in the internal validation cohort; (D) 3-year MACE in the internal validation cohort; (E) 1-year MACE in the external validation cohort; (F) 3-year MACE in the external validation cohort. Abbreviation: MACE= major adverse cardiovascular event. **Table E1**. Demographic data of internal and external set. **Table E2.** Characteristics of patients' medications in three cohorts. **Table E3.** Intra-observer reproducibility of parameters. Abbreviations: CAD-RADS, Coronary Artery Disease - Reporting and Data System; CT-FFR, computed tomography fractional flow reserve; HRP, high-risk plaque; ICC, intraclass correlation coefficient; NRS, napkin-ring sign; PR, positive remodeling; SC, spotty calcification. **Table E4.** Interobserver reproducibility of parameters.

## Data Availability

Data of the current study will be available upon request to corresponding author, if complying with the patient privacy policy of local hospital.

## Abbreviations

CACS: Coronary artery calcium score
CAD: Coronary artery disease
CAD-RADS: Coronary Artery Disease-Reporting and Data System
CCTA: Coronary computed tomography angiography
CT-FFR: Computed tomography angiography-derived fractional flow reserve
HbA1c: Hemoglobin A1c
HRP: High-risk plaque
LAP: Low-attenuation plaque
MACE: Major adverse cardiac event
NRS: Napkin-ring sign
PR: Positive remodeling
SC: Spotty calcification

## References

[1] Glovaci D, Fan W, Wong ND (2019). Epidemiology of diabetes mellitus and cardiovascular disease. Curr Cardiol Rep.

[2] Kronmal RA, McClelland RL, Detrano R, Shea S, Lima JA, Cushman M (2007). Risk factors for the progression of coronary artery calcification in asymptomatic subjects: results from the Multi-Ethnic Study of Atherosclerosis (MESA). Circulation.

[3] Park GM, Lee JH, Lee SW, Yun SC, Kim YH, Cho YR (2015). Comparison of coronary computed tomographic angiographic findings in asymptomatic subjects with versus without diabetes mellitus. Am J Cardiol.

[4] Kedhi E, Berta B, Roleder T, Hermanides RS, Fabris E, AJJ IJ, et al. Thin-cap fibroatheroma predicts clinical events in diabetic patients with normal fractional flow reserve: the COMBINE OCT-FFR trial. Eur Heart J. 2021;42:4671–4679. 10.1093/eurheartj/ehab433 34345911

[5] Zhang W, Singh S, Liu L, Mohammed AQ, Yin G, Xu S (2022). Prognostic value of coronary microvascular dysfunction assessed by coronary angiography-derived index of microcirculatory resistance in diabetic patients with chronic coronary syndrome. Cardiovasc Diabetol.

[6] Gulati M, Levy PD, Mukherjee D, Amsterdam E, Bhatt DL, Birtcher KK (2021). 2021 AHA/ACC/ASE/CHEST/SAEM/SCCT/SCMR guideline for the evaluation and diagnosis of chest pain: a report of the American College of Cardiology/American Heart Association Joint Committee on Clinical Practice Guidelines. Circulation.

[7] Knuuti J, Wijns W, Saraste A, Capodanno D, Barbato E, Funck-Brentano C (2020). 2019 ESC Guidelines for the diagnosis and management of chronic coronary syndromes. Eur Heart J.

[8] Min JK, Shaw LJ, Devereux RB, Okin PM, Weinsaft JW, Russo DJ (2007). Prognostic value of multidetector coronary computed tomographic angiography for prediction of all-cause mortality. J Am Coll Cardiol.

[9] Motoyama S, Sarai M, Harigaya H, Anno H, Inoue K, Hara T (2009). Computed tomographic angiography characteristics of atherosclerotic plaques subsequently resulting in acute coronary syndrome. J Am Coll Cardiol.

[10] Otsuka K, Fukuda S, Tanaka A, Nakanishi K, Taguchi H, Yoshikawa J (2013). Napkin-ring sign on coronary CT angiography for the prediction of acute coronary syndrome. JACC Cardiovasc Imaging.

[11] Cury RC, Abbara S, Achenbach S, Agatston A, Berman DS, Budoff MJ, et al. CAD-RADS(TM) Coronary Artery Disease—Reporting and Data System. An expert consensus document of the Society of Cardiovascular Computed Tomography (SCCT), the American College of Radiology (ACR) and the North American Society for Cardiovascular Imaging (NASCI). Endorsed by the American College of Cardiology. J Cardiovasc Comput Tomogr. 2016;10:269–281. 10.1016/j.jcct.2016.04.005 27318587

[12] Bittner DO, Mayrhofer T, Budoff M, Szilveszter B, Foldyna B, Hallett TR, et al. Prognostic value of coronary CTA in stable chest pain. JACC: Cardiovascular Imaging. 2020;13:1534–45. 10.1016/j.jcmg.2019.09.012 PMC7202954 31734213

[13] Ferencik M, Mayrhofer T, Bittner DO, Emami H, Puchner SB, Lu MT (2018). Use of high-risk coronary atherosclerotic plaque detection for risk stratification of patients with stable chest pain: a secondary analysis of the PROMISE randomized clinical trial. JAMA Cardiol.

[14] Abdelrahman KM, Chen MY, Dey AK, Virmani R, Finn AV, Khamis RY (2020). Coronary computed tomography angiography from clinical uses to emerging technologies: JACC state-of-the-art review. J Am Coll Cardiol.

[15] Meijboom WB, Meijs MF, Schuijf JD, Cramer MJ, Mollet NR, van Mieghem CA (2008). Diagnostic accuracy of 64-slice computed tomography coronary angiography: a prospective, multicenter, multivendor study. J Am Coll Cardiol.

[16] Min JK, Leipsic J, Pencina MJ, Berman DS, Koo BK, van Mieghem C (2012). Diagnostic accuracy of fractional flow reserve from anatomic CT angiography. JAMA.

[17] Norgaard BL, Leipsic J, Gaur S, Seneviratne S, Ko BS, Ito H (2014). Diagnostic performance of noninvasive fractional flow reserve derived from coronary computed tomography angiography in suspected coronary artery disease: the NXT trial (Analysis of Coronary Blood Flow Using CT Angiography: Next Steps). J Am Coll Cardiol.

[18] Douglas PS, De Bruyne B, Pontone G, Patel MR, Norgaard BL, Byrne RA (2016). 1-year outcomes of FFRCT-guided care in patients with suspected coronary disease: the PLATFORM study. J Am Coll Cardiol.

[19] Fairbairn TA, Nieman K, Akasaka T, Norgaard BL, Berman DS, Raff G (2018). Real-world clinical utility and impact on clinical decision-making of coronary computed tomography angiography-derived fractional flow reserve: lessons from the ADVANCE Registry. Eur Heart J.

[20] Norgaard BL, Terkelsen CJ, Mathiassen ON, Grove EL, Botker HE, Parner E (2018). Coronary CT angiographic and flow reserve-guided management of patients with stable ischemic heart disease. J Am Coll Cardiol.

[21] Genders TS, Steyerberg EW, Alkadhi H, Leschka S, Desbiolles L, Nieman K (2011). A clinical prediction rule for the diagnosis of coronary artery disease: validation, updating, and extension. Eur Heart J.

[22] Yu Y, Ding X, Yu L, Dai X, Wang Y, Zhang J (2022). Increased coronary pericoronary adipose tissue attenuation in diabetic patients compared to non-diabetic controls: a propensity score matching analysis. J Cardiovasc Comput Tomogr.

[23] Yu Y, Ding X, Yu L, Lan Z, Wang Y, Zhang J. Prediction of microvascular complications in diabetic patients without obstructive coronary stenosis based on peri-coronary adipose tissue attenuation model. Eur Radiol. 2022. 10.1007/s00330-022-09176-6 36255489

[24] Lu G, Ye W, Ou J, Li X, Tan Z, Li T (2021). Coronary computed tomography angiography assessment of high-risk plaques in predicting acute coronary syndrome. Front Cardiovasc Med.

[25] Cury RC, Abbara S, Achenbach S, Agatston A, Berman DS, Budoff MJ (2016). CAD-RADS™: coronary artery disease—reporting and data system. J Am Coll Radiol.

[26] Li Z, Zhang J, Xu L, Yang W, Li G, Ding D (2020). Diagnostic accuracy of a fast computational approach to derive fractional flow reserve from coronary CT angiography. JACC Cardiovasc Imaging.

[27] Westra J, Li Z, Rasmussen LD, Winther S, Li G, Nissen L (2021). One-step anatomic and function testing by cardiac CT versus second-line functional testing in symptomatic patients with coronary artery stenosis: head-to-head comparison of CT-derived fractional flow reserve and myocardial perfusion imaging. EuroIntervention.

[28] Nozaki YO, Fujimoto S, Kawaguchi YO, Aoshima C, Kamo Y, Sato H (2022). Prognostic value of the optimal measurement location of on-site CT-derived fractional flow reserve. J Cardiol.

[29] Rawshani A, Rawshani A, Franzen S, Eliasson B, Svensson AM, Miftaraj M (2017). Mortality and cardiovascular disease in type 1 and type 2 diabetes. N Engl J Med.

[30] Plante TB, Juraschek SP, Zakai NA, Tracy RP, Cushman M (2019). Comparison of frequency of atherosclerotic cardiovascular disease events among primary and secondary prevention subgroups of the systolic blood pressure intervention trial. Am J Cardiol.

[31] Stevens SR, Segar MW, Pandey A, Lokhnygina Y, Green JB, McGuire DK (2022). Development and validation of a model to predict cardiovascular death, nonfatal myocardial infarction, or nonfatal stroke in patients with type 2 diabetes mellitus and established atherosclerotic cardiovascular disease. Cardiovasc Diabetol.

[32] Xu S, Coleman RL, Wan Q, Gu Y, Meng G, Song K (2022). Risk prediction models for incident type 2 diabetes in Chinese people with intermediate hyperglycemia: a systematic literature review and external validation study. Cardiovasc Diabetol.

[33] Yahagi K, Kolodgie FD, Lutter C, Mori H, Romero ME, Finn AV (2017). Pathology of human coronary and carotid artery atherosclerosis and vascular calcification in diabetes mellitus. Arterioscler Thromb Vasc Biol.

[34] Kim U, Leipsic JA, Sellers SL, Shao M, Blanke P, Hadamitzky M (2018). Natural history of diabetic coronary atherosclerosis by quantitative measurement of serial coronary computed tomographic angiography: results of the PARADIGM study. JACC Cardiovasc Imaging.

[35] Halon DA, Lavi I, Barnett-Griness O, Rubinshtein R, Zafrir B, Azencot M (2019). Plaque morphology as predictor of late plaque events in patients with asymptomatic type 2 diabetes: a long-term observational study. JACC Cardiovasc Imaging.

[36] Blanke P, Naoum C, Ahmadi A, Cheruvu C, Soon J, Arepalli C (2016). Long-term prognostic utility of coronary CT angiography in stable patients with diabetes mellitus. JACC Cardiovasc Imaging.

[37] Cook CM, Petraco R, Shun-Shin MJ, Ahmad Y, Nijjer S, Al-Lamee R (2017). Diagnostic accuracy of computed tomography-derived fractional flow reserve : a systematic review. JAMA Cardiol.

[38] Garcia-Garcia HM, Klauss V, Gonzalo N, Garg S, Onuma Y, Hamm CW (2012). Relationship between cardiovascular risk factors and biomarkers with necrotic core and atheroma size: a serial intravascular ultrasound radiofrequency data analysis. Int J Cardiovasc Imaging.

[39] Kay-Tee Khaw M, Nicholas Wareham, MBBS, Sheila Bingham, Robert Luben, et al. Association of hemoglobin a1c with cardiovascular disease and mortality in adults: the european prospective investigation into cancer in norfolk. Ann Intern Med. 2004;141:413–20. 10.7326/0003-4819-141-6-200409210-00006 15381514

[40] Ronald K. Hyperglycemia and microvascular and macrovascular disease in diabetes. Diabetes Care. 1995;18(2):258–68. 10.2337/diacare.18.2.258 7729308

[41] Cahn A, Wiviott SD, Mosenzon O, Goodrich EL, Murphy SA, Yanuv I (2022). Association of baseline HbA1c with cardiovascular and renal outcomes: analyses from DECLARE-TIMI 58. Diabetes Care.

[42] Stratton IM, H Andrew, W Neil, DR Matthews, SE Manley, CA Cull, et al. Association of glycaemia with macrovascular and microvascular complications of type 2 diabetes (UKPDS 35): prospective observational study. BMJ. 2000;321(7258):405–12. 10.1136/bmj.321.7258.405 PMC27454 10938048

[43] Jiang L, Shi K, Guo YK, Ren Y, Li ZL, Xia CC (2020). The additive effects of obesity on myocardial microcirculation in diabetic individuals: a cardiac magnetic resonance first-pass perfusion study. Cardiovasc Diabetol.

[44] Coenen A, Rossi A, Lubbers MM, Kurata A, Kono AK, Chelu RG (2017). Integrating CT myocardial perfusion and CT-FFR in the work-up of coronary artery disease. JACC Cardiovasc Imaging.

