# Supplementary material for: CT-derived fractional flow reserve for prediction of major adverse cardiovascular events in diabetic patients
Source: Cardiovasc Diabetol. 2023 Mar 21;22:65. doi: 10.1186/s12933-023-01801-y (PMC10032006; doi:10.1186/s12933-023-01801-y)
Supplement: Supplementary file 1 — Additional file 1. Figure E1. Kaplan–Meier curves for patients in the low- and high-risk groups in training cohort (A), internal validation cohort (B), and external validation cohort (C). Figure E2. Decision curves of prediction model for (A) 1-year MACE in the training cohort; (B) 3-year MACE in the training cohort internal; (C) 1-year MACE in the internal validation cohort; (D) 3-year MACE in the internal validation cohort; (E) 1-year MACE in the external validation cohort; (F) 3-year MACE in the external validation cohort. Abbreviation: MACE= major adverse cardiovascular event. Table E1. Demographic data of internal and external set. Table E2. Characteristics of patients' medications in three cohorts. Table E3. Intra-observer reproducibility of parameters. Abbreviations: CAD-RADS, Coronary Artery Disease - Reporting and Data System; CT-FFR, computed tomography fractional flow reserve; HRP, high-risk plaque; ICC, intraclass correlation coefficient; NRS, napkin-ring sign; PR, positive remodeling; SC, spotty calcification. Table E4. Interobserver reproducibility of parameters. [file 12933_2023_1801_MOESM1_ESM.docx]

***Supplementary appendix***

**CCTA acquisition**

A third-generation dual source CT (SOMATOM Force, Siemens Healthineers, Germany) or a 256-slice wide detector CT scanner (Revolution HD, GE Healthcare, USA) was used for scanning. Calcium score was firstly performed before the acquisition of CCTA to assess the calcification burden of each coronary branch. CCTA was performed using a bolus tracking technique, with the region of interest located in the ascending aorta. All patients received a bolus of a contrast media (Iomeprol, Iomeron, 400 mg iodine/ml, Bracco, Italy) into the antecubital vein at a rate of 4-5 ml/s, followed by flushing of 40 ml of saline using a dual-barrel power injector. The amount of the contrast media was determined based on the patient's body weight and the scanning time. Tube voltage and tube current were automatically adjusted by the automatic exposure control system on both scanners (CAREKv, CAREDose 4D, Siemens Healthineers, Germany on third-generation dual source CT; KV Assist, Smart mA, GE Healthcare, USA on 256-slice wide detector CT scanner) according to the patient's body habitus. CCTA images were acquired using a prospective ECG-trigger sequence, and the acquisition window covered 35%-75% of the R-R interval. For third-generation dual source CT, the reconstruction parameters of CCTA images were as follows: collimation = 96×0.6 mm, reconstructed slice thickness = 0.75 mm, reconstructed slice interval = 0.5mm, rotation time = 250 ms. The reference tube current was set as 320 mAs and the reference tube voltage was set as 100 kVp. For 256-slice wide detector CT scanner, the reconstruction parameters of CCTA images were as follows: collimation = 256×0.625 mm, reconstructed slice thickness = 0.625 mm, reconstructed slice interval = 0.5mm, rotation time = 280 ms.

**CT-FFR calculation**

CT-FFR simulation was performed on a prototype software package (Cta-Plus; version 2.0, Pulse Medical Imaging Technology, China) based on quantitative flow ratio (CT-QFR) technology. For on-site processing, firstly the software automatically depicted the coronary vessels and all epicardial vessels ≥ 2 mm in diameter were analyzed. Secondly, all coronary arteries were segmented and combined into a hierarchical tree structure and the reference lumen (the normal lumen without atherosclerosis) was reconstructed as if there were no stenosis. Subsequently, patient-specific resting coronary blood flow was calculated based on its associated reference coronary lumen size and the anisotropic scaling law, which was then converted to virtual congested blood flow. Finally, CT-FFR values were calculated for each location of the reconstructed coronary tree using the virtual congestion flow using the previously validated QFR algorithm. Manual adjustment of lumen segmentation was available if needed to ensure correct CT-FFR simulation.

Lesion-specific CT-FFR value was measured 1-2 cm distal to the lesion for all coronary stenosis on major epicardial vessels with diameter ≥ 2mm. Vessel-specific CT-FFR was defined as the CT-FFR value for the most distal lesion. For vessels without significant stenosis, the CT-FFR value was recorded at the most distal site where vessel diameter was ≥ 2mm. The lowest vessel-specific CT-FFR value of major epicardial arteries was used for patient-based analysis and the presence of any vessel-specific CT-FFR ≤ 0.80 was considered hemodynamically significant.

Two cardiovascular radiologists (with 3-years and 12-years experience of cardiac imaging), who were blinded to the clinical history and outcomes, independently analyzed the lesions. The mean CT-FFR value of measurement by two observers was recorded for further analysis.

Supplemental Table E1 Demographic data of internal and external set.

|  | **Total** | **Internal set** | **External set** | **p** |
| --- | --- | --- | --- | --- |
|  | ***N=1797*** | ***N=1368*** | ***N=429*** |  |
| Age(years) | 61.0 [54.0-69.0] | 62.0 [55.0-69.0] | 61.0 [54.0-68.0] | 0.061 |
| Males, n (%) | 1031 (57.4) | 779 (56.9) | 252 (58.7) | 0.548 |
| BMI, kg/m^2^ | 24.5 [22.5-26.8] | 24.4 [22.4-26.6] | 25.0 [22.6-27.3] | 0.008 |
| Course of diabetes(years) | 10.0 [3.00-16.0] | 10.0 [4.00-16.0] | 10.0 [3.00-16.0] | 0.630 |
| Hypertension, n (%) | 993 (55.3) | 751 (54.9) | 242 (56.4) | 0.621 |
| Dyslipidemia, n (%) | 1104 (61.4) | 792 (57.9) | 312 (72.7) | <0.001 |
| Current smoking, n (%) | 550 (30.6) | 422 (30.8) | 128 (29.8) | 0.737 |
| Fast glucose(mmol/L) | 7.10 [5.60-9.80] | 6.90 [5.50-9.50] | 7.67 [6.00-10.8] | <0.001 |
| HbA1c (%) | 8.40 [7.20-10.1] | 8.40 [7.20-10.0] | 8.50 [7.20-10.3] | 0.386 |
| Radiation dose, mSv | 1.98 [1.31-3.02] | 1.89 [1.22-2.59] | 2.49 [1.70-4.85] | <0.001 |
| CT-FFR≤0.80, n (%) | 140 (7.79) | 114 (8.33) | 26 (6.06) | 0.153 |
| Obstructive CAD, n (%) | 503 (28.0) | 413 (30.2) | 90 (21.0) | <0.001 |
| CAD-RADS, n (%): |  |  |  | . |
| 0 | 473 (26.3) | 334 (24.4) | 139 (32.4) |  |
| 1 | 395 (22.0) | 294 (21.5) | 101 (23.5) |  |
| 2 | 426 (23.7) | 327 (23.9) | 99 (23.1) |  |
| 3 | 306 (17.0) | 247 (18.1) | 59 (13.8) |  |
| 4A | 173 (9.63) | 146 (10.7) | 27 (6.29) |  |
| 4B | 19 (1.06) | 17 (1.24) | 2 (0.47) |  |
| 5 | 5 (0.28) | 3 (0.22) | 2 (0.47) |  |
| CACS n (%): |  |  |  | 0.976 |
| 0 | 785 (43.7) | 594 (43.4) | 191 (44.5) |  |
| 0-100 | 664 (37.0) | 507 (37.1) | 157 (36.6) |  |
| 100-400 | 229 (12.7) | 175 (12.8) | 54 (12.6) |  |
| >400 | 119 (6.62) | 92 (6.73) | 27 (6.29) |  |
| HRP, n (%) | 491 (27.3) | 390 (28.5) | 101 (23.5) | 0.051 |
| LAP, n (%) | 494 (27.5) | 397 (29.0) | 97 (22.6) | 0.011 |
| PR, n (%) | 956 (53.2) | 731 (53.4) | 225 (52.4) | 0.762 |
| SC, n (%) | 108 (6.01) | 85 (6.21) | 23 (5.36) | 0.595 |
| NRS, n (%) | 170 (9.46) | 142 (10.4) | 28 (6.53) | 0.022 |
| Microvascular complications, n (%) | 1312 (73.0) | 982 (71.8) | 330 (76.9) | 0.042 |
| MACE, n (%) | 129 (7.18) | 100 (7.31) | 29 (6.76) | 0.781 |

P: the p between Training set + Internal validation set and External validation set.

Values are mean ± SD, n (%), or median (IQR). SD, standard deviation.

Abbreviations: BMI, body mass index; CACS, Coronary Artery Calcium Scoring; CAD, Coronary artery disease; CAD-RADS, Coronary Artery Disease - Reporting and Data System; CT-FFR, computed tomography fractional flow reserve; HbA1c, hemoglobin A1c; HRP, high-risk plaque; LAP, low-attenuation plaque; MACE, major adverse cardiac events. NRS, napkin-ring sign; PR, positive remodeling; SC, spotty calcification.

Supplemental Table E2. Characteristics of patients' medications in three cohorts

|  | **Training set** | | | **Internal validation set** | | | | **External validation set** | | | |
| --- | --- | --- | --- | --- | --- | --- | --- | --- | --- | --- | --- |
|  | **MACE (-)** | **MACE (+)** | **p value** | **MACE (-)** | **MACE (+)** | **p value** | **MACE (-)** | | **MACE (+)** | **p value** |  |
|  | ***N=892*** | ***N=65*** |  | ***N=376*** | ***N=35*** |  | ***N=400*** | | ***N=29*** |  |  |
| Hypoglycemic medications |  |  |  |  |  |  |  | |  |  |  |
| Insulin secretagogues, n (%) | 263 (29.5) | 14 (21.5) | 0.222 | 103 (27.4) | 11 (31.4) | 0.755 | 113 (28.2) | | 3 (10.3) | 0.060 |  |
| TZDs, n (%) | 18 (2.02) | 2 (3.08) | 0.641 | 8 (2.13) | 1 (2.86) | 0.555 | 5 (1.25) | | 2 (6.90) | 0.075 |  |
| Insulin, n (%) | 377 (42.3) | 26 (40.0) | 0.821 | 148 (39.4) | 17 (48.6) | 0.377 | 190 (47.5) | | 18 (62.1) | 0.186 |  |
| Biguanides, n (%) | 437 (49.0) | 30 (46.2) | 0.754 | 172 (45.7) | 19 (54.3) | 0.428 | 204 (51.0) | | 20 (69.0) | 0.093 |  |
| α-Glucosidase inhibitors, n (%) | 288 (32.3) | 29 (44.6) | 0.057 | 116 (30.9) | 12 (34.3) | 0.819 | 131 (32.8) | | 7 (24.1) | 0.452 |  |
| DPP-4 inhibitors, n (%) | 238 (26.7) | 22 (33.8) | 0.267 | 100 (26.6) | 9 (25.7) | 1.000 | 139 (34.8) | | 9 (31.0) | 0.838 |  |
| SGLT-2 inhibitors, n (%) | 31 (3.48) | 3 (4.62) | 0.498 | 17 (4.52) | 1 (2.86) | 1.000 | 11 (2.75) | | 3 (10.3) | 0.061 |  |
| GLP-1 RAs, n (%) | 32 (3.59) | 3 (4.62) | 0.727 | 11 (2.93) | 1 (2.86) | 1.000 | 12 (3.00) | | 1 (3.45) | 0.603 |  |
| Antihypertensive agents |  |  |  |  |  |  |  | |  |  |  |
| β-blockers, n (%) | 48 (5.38) | 4 (6.15) | 0.775 | 24 (6.38) | 6 (17.1) | 0.032 | 11 (2.75) | | 3 (10.3) | 0.061 |  |
| ACEI, n (%) | 59 (6.61) | 6 (9.23) | 0.439 | 28 (7.45) | 4 (11.4) | 0.336 | 17 (4.25) | | 5 (17.2) | 0.012 |  |
| ARB, n (%) | 236 (26.5) | 19 (29.2) | 0.732 | 93 (24.7) | 10 (28.6) | 0.766 | 117 (29.2) | | 8 (27.6) | 1.000 |  |
| Ca2+ channel blockers, n (%) | 265 (29.7) | 19 (29.2) | 1.000 | 92 (24.5) | 16 (45.7) | 0.011 | 112 (28.0) | | 14 (48.3) | 0.035 |  |
| Statins, n (%) | 104 (11.7) | 12 (18.5) | 0.154 | 58 (15.4) | 7 (20.0) | 0.640 | 72 (18.0) | | 9 (31.0) | 0.137 |  |

Values are mean ± SD, n (%), or median (IQR). SD, standard deviation.

Abbreviations: ACEI= angiotensin-converting enzyme inhibitors; ARB= angiotensin receptor antagonist; DPP-4= dipeptidyl peptidase 4; GLP-1 RA= glucagon-like peptide 1 receptor agonist; SGLT-2= sodium-glucose cotransporter 2; TZDs= Thiazolidinedione drugs.

Supplemental Table E3. Intra-observer reproducibility of parameters

|  | ICC (95% CI) | P value |
| --- | --- | --- |
| CT-FFR | 0.978(0.971-0.983) | < 0.001 |
| Any HRP | 0.944(0.927-0.957) | < 0.001 |
| Any LAP | 0.931(0.909-0.947) | < 0.001 |
| Any NRS | 0.960(0.948-0.970) | < 0.001 |
| Any SC | 0.939(0.920-0.953) | < 0.001 |
| Any PR | 0.961(0.949-0.970) | < 0.001 |
| CAD-RADS | 0.879(0.843-0.907) | <0.001 |

Abbreviations: CAD-RADS, Coronary Artery Disease - Reporting and Data System; CT-FFR, computed tomography fractional flow reserve; HRP, high-risk plaque; ICC, intraclass correlation coefficient; NRS, napkin-ring sign; PR, positive remodeling; SC, spotty calcification

Supplemental Table E4. Interobserver reproducibility of parameters

|  | ICC (95% CI) | P value |
| --- | --- | --- |
| CT-FFR | 0.947(0.931-0.960) | < 0.001 |
| Any HRP | 0.917(0.891-0.936) | < 0.001 |
| Any LAP | 0.903(0.873-0.925) | < 0.001 |
| Any NRS | 0.919(0.894-0.938) | < 0.001 |
| Any SC | 0.871(0.833-0.901) | < 0.001 |
| Any PR | 0.933(0.912-0.949) | < 0.001 |
| CAD-RADS | 0.839(0.793-0.876) | <0.001 |

Abbreviations: CAD-RADS, Coronary Artery Disease - Reporting and Data System; CT-FFR, computed tomography fractional flow reserve; HRP, high-risk plaque; ICC, intraclass correlation coefficient; NRS, napkin-ring sign; PR, positive remodeling; SC, spotty calcification


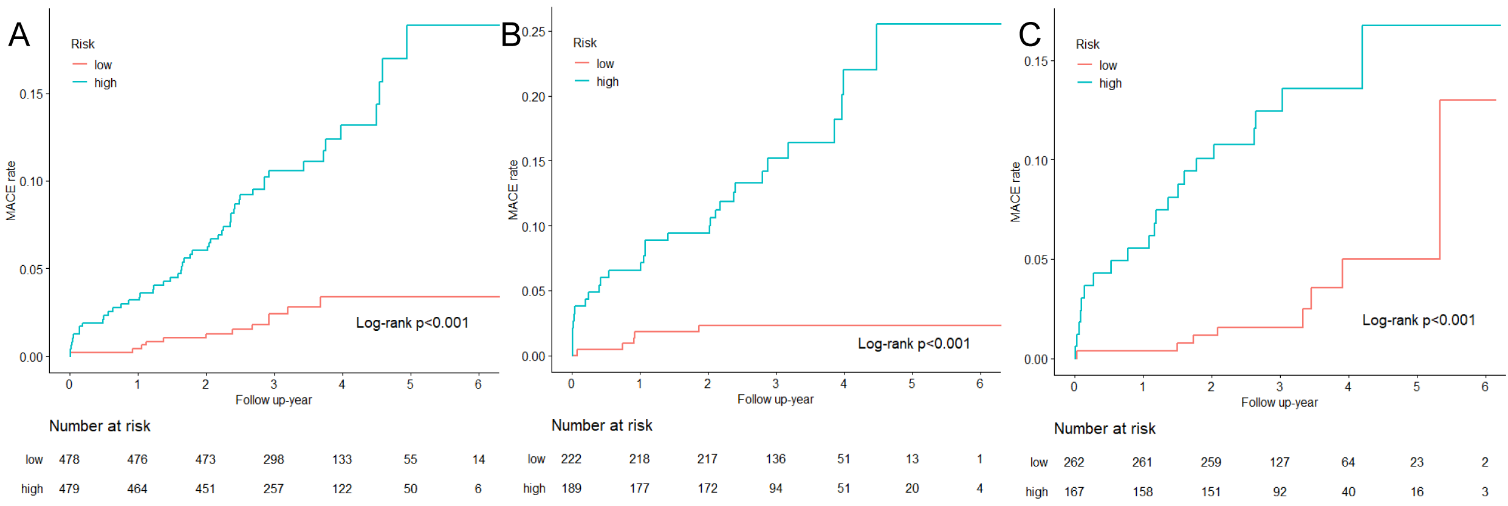


Supplemental Figure E1 Kaplan–Meier curves for patients in the low- and high-risk groups in training cohort (A), internal validation cohort (B), and external validation cohort (C).


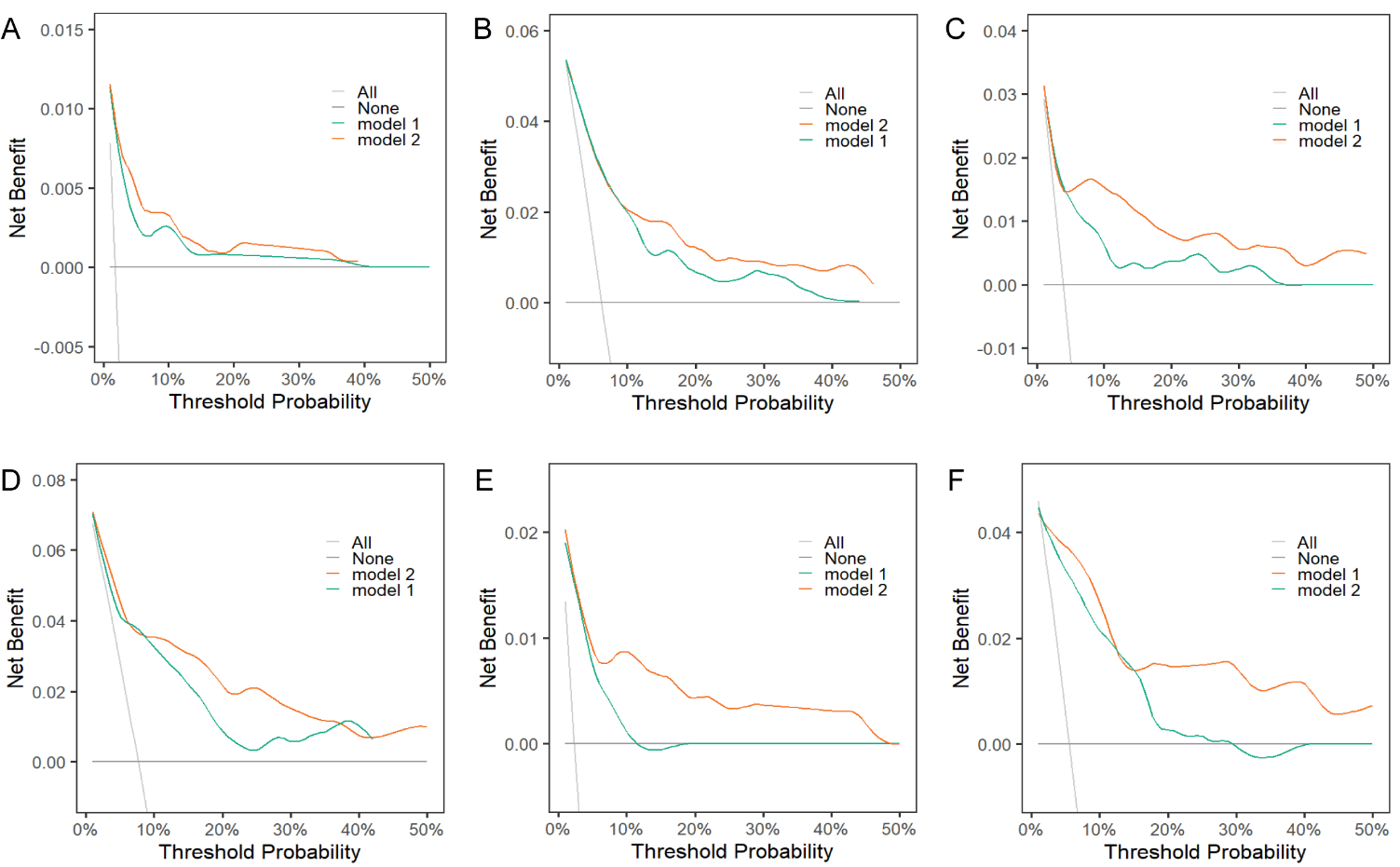


Supplemental Figure E2 Decision curves of prediction model for (A) 1-year MACE in the training cohort; (B) 3-year MACE in the training cohort internal; (C) 1-year MACE in the internal validation cohort; (D) 3-year MACE in the internal validation cohort; (E) 1-year MACE in the external validation cohort; (F) 3-year MACE in the external validation cohort.

Abbreviation: MACE= major adverse cardiovascular event
